# Microbial Contaminants of Cord Blood Units Identified by 16S rRNA Sequencing and by API Test System, and Antibiotic Sensitivity Profiling

**DOI:** 10.1371/journal.pone.0141152

**Published:** 2015-10-29

**Authors:** Luís França, Catarina Simões, Marco Taborda, Catarina Diogo, Milton S. da Costa

**Affiliations:** 1 Center for Neuroscience and Cell Biology, University of Coimbra, Coimbra, Portugal; 2 Microbiology Unit, BIOCANT Biotechnological Park, Cantanhede, Portugal; Wilfrid Laurier University, CANADA

## Abstract

Over a period of ten months a total of 5618 cord blood units (CBU) were screened for microbial contamination under routine conditions. The antibiotic resistance profile for all isolates was also examined using ATB strips. The detection rate for culture positive units was 7.5%, corresponding to 422 samples.16S rRNA sequence analysis and identification with API test system were used to identify the culturable aerobic, microaerophilic and anaerobic bacteria from CBUs. From these samples we recovered 485 isolates (84 operational taxonomic units, OTUs) assigned to the classes *Bacteroidia*, *Actinobacteria*, *Clostridia*, *Bacilli*, *Betaproteobacteria* and primarily to the *Gammaproteobacteria*. Sixty-nine OTUs, corresponding to 447 isolates, showed 16S rRNA sequence similarities above 99.0% with known cultured bacteria. However, 14 OTUs had 16S rRNA sequence similarities between 95 and 99% in support of genus level identification and one OTU with 16S rRNA sequence similarity of 90.3% supporting a family level identification only. The phenotypic identification formed 29 OTUs that could be identified to the species level and 9 OTUs that could be identified to the genus level by API test system. We failed to obtain identification for 14 OTUs, while 32 OTUs comprised organisms producing mixed identifications. Forty-two OTUs covered species not included in the API system databases. The API test system Rapid ID 32 Strep and Rapid ID 32 E showed the highest proportion of identifications to the species level, the lowest ratio of unidentified results and the highest agreement to the results of 16S rRNA assignments. Isolates affiliated to the *Bacilli* and *Bacteroidia* showed the highest antibiotic multi-resistance indices and microorganisms of the *Clostridia* displayed the most antibiotic sensitive phenotypes.

## Introduction

Over the last three decades, public and private cord blood banks have been established worldwide to cryopreserve stem cells for long periods of time with the premise that they can be used later for the treatment of a variety of hematologic disorders and diseases in children and adults [1].

Despite the guidelines recommended by international standards for collecting and processing cord blood units (CBUs), the literature reports a significant risk of microbial contamination during the retrieval and separation of blood cells from umbilical cords [2]. To this end, NetCord-FACT International Standards for Cord Blood Collection mandates the microbial screening, prior to cryopreservation of the post-processed CBUs, using a system validated for the growth of aerobic and anaerobic bacteria and fungi. Microorganisms isolated from the CBUs should include identification and antibiotic sensitivity testing of the organisms [3].

Clinical laboratories rely mainly on standardized phenotypic test systems coupled with culture- and microscopy-based methods for the identification of microorganisms with clinical importance [4]. These methods permit the identification of most clinical isolates with great accuracy and simplicity, but they are costly and time-consuming. Also, they suffer from several shortcomings such as limited databases that do not comprise all bacterial species or infrequent biotypes, intraspecies phenotypic variability and ambiguous phenotypic profiles [4]. The analysis of the 16S rRNA genes has been one of the most valuable alternatives in providing the identification of isolates with deviant biochemical profiles or for taxa that are rarely associated with human infectious diseases. Difficulties with the identification associated with the lack of a clear similarity threshold value for the rank of species and the overall quality of nucleotide sequences deposited in public databases, which is sometimes questionable, hamper the application of 16S rRNA analysis as an exclusive approach for clinical microbial identification [5].

In this study, we analyzed a total of 5618 CBUs during a period of ten months, with the recovery of 422 samples positive for microorganisms (7.5% of total CBUs). Our objectives were i) to identify the most common microbial populations that contaminate CBUs in a cord blood bank, ii) to compare the performance of a standardized phenotypic test system (API, Biomerieux) in identifying CBU isolates with 16S rRNA sequencing and iii) to determine the diversity of antibiotic resistant profiles for the bacterial species recovered.

## Methods

### Screening of CBUs and isolation of microorganisms

CBUs are processed and cryopreserved by Crioestaminal (www.crioestaminal.pt), a cord blood facility located in Central Portugal. The plasma of the cord blood samples is routinely screened for microbial contamination prior to cryopreservation in liquid nitrogen. Samples obtained during April 2010 to January 2011 were used in this investigation (5618 CBUs). As this was an observational study, requiring non invasive procedures of assessment, no informed consent was required as the data were analyzed anonymously. All donors provided a consent form for Crioestaminal to process, store the CBUs and perform these analysis with the samples. No author had knowledge of the identity of the donors.

The blood was recovered from the umbilical cords by the obstetrician into single blood bag systems for collection of cord blood (150 ml, containing 21 ml of CPDA-1 anticoagulant, Obelis, S. A., Brussels, Belgium) and sent by courier delivery to the cord blood bank. The blood and plasma were aseptically separated from stem cells at the blood bank, before the plasma was sent for microbiological quality control. This process took 24 to 72 hours after collection of the blood from the umbilical cords with bags being maintained at 18–22°C.

Five ml of umbilical cord plasma was inoculated, using disposable seringes (10 ml), into BacT/ALERT SA (aerobic) and BacT/ALERT SN (anaerobic) culture bottles (BioMérieux, Durham, NC, USA) and placed in the BacT/ALERT 3D system incubator at 35°C for a maximum of 7 days or until they were flagged as positive. Negative controls consisting of SA and SN bottles were injected with 5 ml of sterile phosphate buffered saline with every batch of fifty samples. A volume of 100 μl of each positive culture bottle was used to inoculate Columbia agar plates supplemented with 5% horse blood (COH, BioMérieux, Durham, NC, USA). The positive cultures were then spread on the entire Petri plates, and incubated at 35°C for up to 5 days under aerobic, microaerobic (10% CO_2_, Binder CB 150, Tuttlingen, Germany) and anaerobic conditions (GENBox Jar 2.5L, GENbox anaer, BioMérieux, Durham, NC, USA). At least six separate colonies with the same colony morphology were picked from the plates and subcultured on the same medium until considered to have been purified. In some cases colonies overlapped and subculturing had to be performed until separate colonies were obtained. Isolates were grouped by Gram staining and cellular morphology, colony morphology, oxidase and catalase activities. One isolate of each phenotype from each COH plate was selected for further processing and maintained at –80°C in Tryptic Soy Broth (TSB) containing 15% (w/v) glycerol

### Microbial identification using the 16S rRNA sequencing

DNA of representatives of each phenotypic profile was extracted following the procedure of Marmur [6]. The 16S rRNA gene was amplified with primers 27F (5’GAGTTTGATCCTGGCTCAG3’) and 1525R (5’AGAAAGGAGGTGATCCAGCC3’) as previously described [7]. Amplicons were purified using Jetquick PCR purification Kit (Genomed GmbH, Löhne, Germany) and partially sequenced by Macrogen Europe (Macrogen Inc., Seoul, Korea). The quality of the 16S rRNA gene sequences was checked manually using the Sequence Scanner Software V1.0 (Life Technologies Corporation, Carlsbad, CA, USA). 16S rRNA genes were initially grouped based on sequences with the same nucleotide sequence over aligned columns encompassing V1 to V5 hypervariable regions (~800nt). The 16S rRNA gene of representatives of each unique ribotype were almost fully sequenced using the method described above and grouped into Operational Taxonomic Units (OTUs) by clustering sequences with 99.0% sequence similarity or higher using the script pick_otus.py implemented in the QIIME software package [8].

The phylogenetic inference was performed using the ARB software package using the Living Tree Project (LTP) release 115 [9,10]. The LTP dataset was used for the automatic alignment of the sequences using the SINA stand-alone version 1.2.11 [11]. Representative sequences of each OTU were inserted into the LTPs115 tree using the ARB-parsimony tool implemented in the ARB software package [9] and closely related reference sequences were selected. A *de novo* phylogenetic reconstruction of isolates using almost full length sequences with the reference sequences was calculated using the RAxML algorithm with the GTRGAMMA model [12], with a 20% maximum frequency filter to remove noise and preserve positional orthology. Taxonomic assignment of each OTU was performed based on the classification of taxon thresholds for the species of ≥99.0%, genus between <99.0% and ≥94.5%, family <94.5.0% and ≥86.5% [13,14]. Shannon—Wiener, Dominance-D, Chao1 indices and rarefaction analysis were calculated using the PAST software v1.82b [15].

The 16S rRNA gene sequences of representatives of each OTU were deposited in GenBank under the accession numbers KR232840-KR232923.

### Microbial identification using the API test system

An appropriate API strip (BioMérieux) for the identification of each broad phenotypic category was selected following the manufacturer’s instructions. Briefly, the rapid ID 32 E or ID 32 GN was used to identify Gram-negative rods; the ID 32 Staph system was used for Gram-positive cocci as well as other catalase-positive organisms, while the Rapid ID 32 Strep system was used to identify Gram-positive catalase negative cocci. The API Coryne or the API 50 CHL strips was used to identify Gram-positive catalase positive or catalase negative rods; the API 50 CHB strips was used for Gram-positive spore-producing rods while the Rapid ID 32A was used for the identification of all anaerobic isolates.

Bacterial suspensions were prepared from fresh cultures grown on COH. Inoculation and incubation of API strips were performed following the manufacturer’s instructions. The miniAPI Analyser (BioMérieux) was used to record all results using the database version 1.5.2/INT08. Only results reported as “acceptable, good, very good and excellent” were used for identification at the genus and the species level. Results reported as “poor discrimination, unacceptable and not interpretable” were recorded as “unidentified” after repeated testing. Bacterial nomenclature of API system databases were checked against the Approved Lists of Bacterial Names (http://www.bacterio.net/) and corrected, if necessary.

### Antimicrobial susceptibility testing

Isolates were tested for antimicrobial susceptibility using ATB strips (BioMérieux) coupled with the miniAPI Analyzer to detect turbidity indicative of microbial growth in the wells with different antibiotics. Appropriate ATB strip were chosen as recommended by the manufacturer. Briefly, ATB STAPH5 or ATB STAPHEU were used for staphylococci-like microorganisms, ATB G-5 for enterobacteria and other cytochrome oxidase negative Gram-negative rods, ATB PSE5 for *Pseudomonas* spp. and other cytochrome oxidase positive Gram-negative rods; ATB ENTEROCOC 5 for enterococcal microorganisms, ATB STREP 5 or ATB STREP EU (08) for streptococcal and pneumococcal microorganisms, and ATB ANA or ATB ANA EU (08) for strictly anaerobic bacteria.

To expand the number of isolates assessed we used ATB susceptibility test strips for bacteria not belonging to the above mentioned groups but identified with the API systems, namely ATB ANA or ATB ANA EU (08) strips were also used to examine antibiotic sensitivity in aerotolerant strains, such as *Lactobacillus* spp., *Propionibacterium* spp. and *Actinomyces* spp. ATB STAPH were used to determine antibiotic sensitivity in catalase-positive Gram-positive rods, namely *Brevibacterium* spp., *Bacillus* spp., *Corynebacterium* spp. and *Zimmermannella* spp. The ATB ENTEROCOC 5 strips were used for antimicrobial susceptibility evaluation of the *Aerococcus urinae* and *Lactococcus lactis* subsp. *lactis* and ATB STREP EU was used to analyze *Trueperella spp*. with a negative catalase reaction.

ATB test strips were inoculated with microbial suspensions made from fresh cultures and incubated according to manufacturer’s instructions. For each antibiotic, miniAPI Analyzer was used to measure the turbidity in the test wells that were recorded as follows: sensitive, no growth; intermediate, growth only at a low concentration when the antibiotic was tested at two concentrations; or resistant, when growth was observed at both concentrations.

The disk diffusion method was used for strains unable to grow on control wells of ATB test strips. Susceptibility testing was performed on COH plates incubated at 35°C for 24-48h under anaerobic conditions, following described standards [16] and using appropriate controls to attest the effect of COH medium on antibiotic susceptibility. The antibiotics tested were: amoxicillin-clavulanic acid 30μg, penicillin 10μg, piperacillin 30μg, piperacillin-tazobactam 30/6μg, ticarcillin-clavulanic acid 75/10μg, imipenem 10μg; cefotetan 30μg, cefoxitin 30μg, clindamycin 2μg, chloramphenicol 30μg and metronidazol 4μg (all Biorad, USA). *Escherichia coli* ATCC 25922, *Pseudomonas aeruginosa* DSM 1117 and *Bacteroides fragilis* ATCC 23745 were included as controls.

## Results

### Positive CBUs for microbial contamination

A total of 5618 CBUs were screened for aerobic and anaerobic bacteria, and fungal contamination using the BacT/ALERT system. The detection rate for the positive post-processed CBUs was 7.5%, corresponding to 422 samples that became positive over 1 to 6 days (mean 2.5 days). Ten samples yielded false-positive results after confirmatory examination for growth on COH agar plates and were excluded from further analysis. The majority of the positive samples showed bacterial growth in both aerobic and anaerobic BacT/ALERT culture bottles (52.0%). However, 7.3% of the samples were only positive in aerobic bottles and 40.7% were only positive under anaerobic conditions.

### Microbial identification using the API test system and 16S rRNA sequencing

A total of 485 isolates, recovered from the 422 positive samples, were analyzed by primary culture tests, namely Gram reaction, aerobic, anaerobic, facultative anaerobic growth, catalase and cytochrome oxidase, to choose the appropriate API test system to be used and compared to 16S rRNA sequencing. A total of 2 samples were found to contain four different bacterial taxa, seven of the samples had three bacterial taxa and forty-four of the samples had two bacterial taxa. We recovered only one taxon from each of the remaining 368 samples. The large majority of the isolates (60.6%) were facultative anaerobes, 6.7% of the isolates were obligate aerobes, 30% grew only under anaerobic conditions and the remaining 2.8% of the strains grew under anaerobic and microaerophilic conditions.

All isolates were grouped into OTUs defined at ≥ 99.0% sequence similarity of the 16S rRNA gene (≥1270 bp). Alpha diversity calculations indicated a value of 3.51 for Shannon—Wiener index and 0.07 for Dominance-D. Rarefaction curve for the observed OTU numbers fail to reach an asymptote (S1 Fig). Chao1 estimator suggested that there were 12 additional ribotypes that were not isolated.

Based on the phylogenetic inference we recovered 84 OTUs belonging to the classes *Bacteroidia*, *Actinobacteria*, *Clostridia*, *Bacilli*, *Betaproteobacteria* and primarily to the *Gammaproteobacteria* (S1 Table and S2 Fig). Fungi were not detected in any of the samples examined. The majority of the OTUs (69 OTUs), corresponding to 447 isolates, had 16S rRNA sequence similarities above the stringent cut-off value of 99.0% with known cultured microorganisms (S1 Table). Fourteen OTUs (n = 37 isolates) were assigned at the genus level and one OTU (n = 1) at the family level.

**Fig 1:**
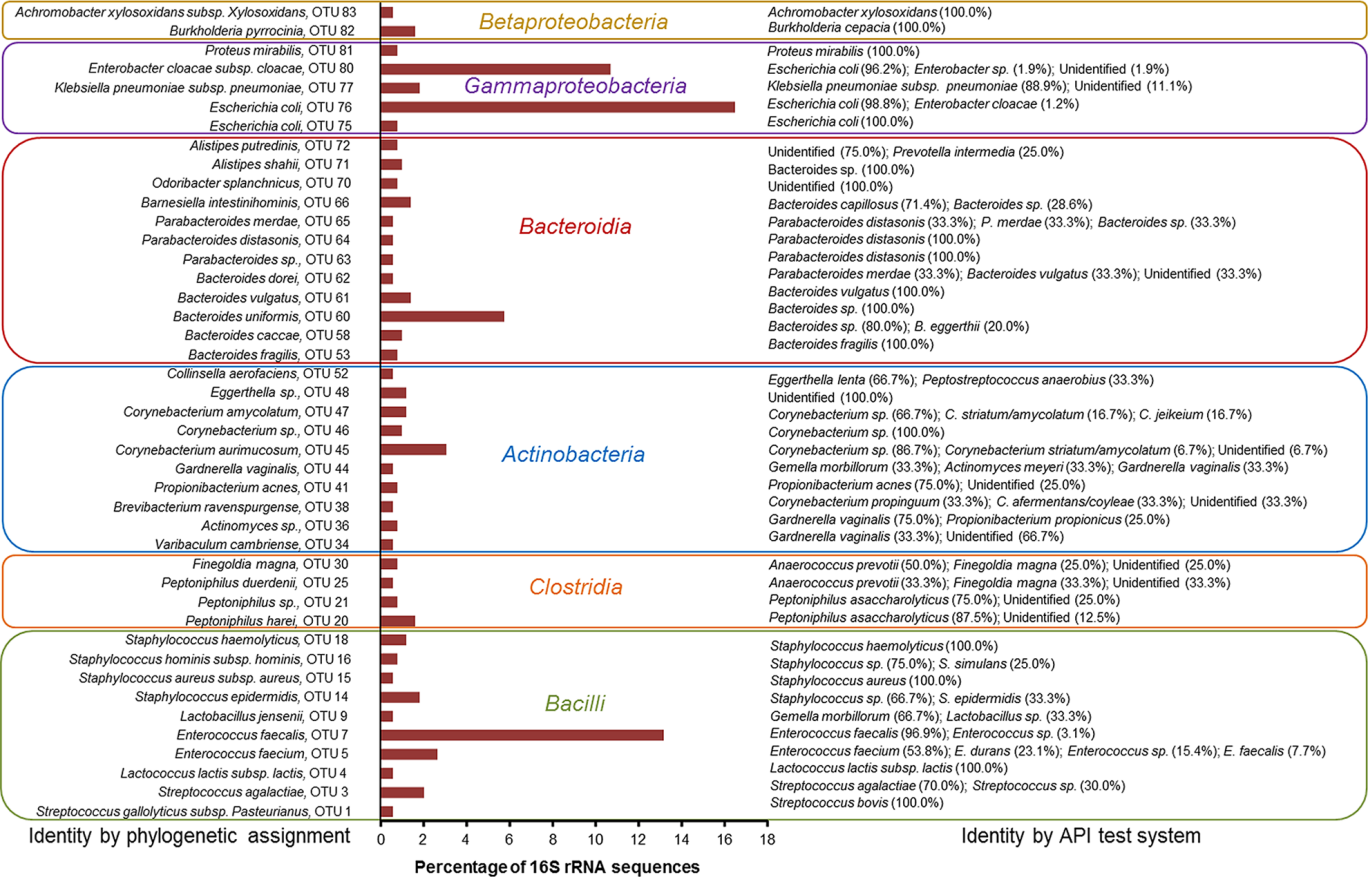
Relative proportions of the different OTUs obtained in CBUs positive for microbial contamination. OTUs were identified by phylogenetic inference of 16S rRNA sequences and by API test system. In some cases, isolates grouped into an OTU showed different results using the API test system, whose relative proportions are shown in brackets. Only OTUs clustering more than 2 isolates are shown.

The Gram-negative population (52.2% of total number of isolates) were composed of 32 OTUs, dominated mainly by members of the *Enterobacteriaceae*, namely *Escherichia coli* (OTU 76) and *Enterobacter cloacae subsp*. *cloacae* (OTU 80), and by *Bacteroides* spp., mainly *B*. *uniformis* (OTU 60) (Fig 1 and S1 Table). Gram-positive bacteria were more diverse (52 OTUs) being composed mostly by representatives of the genera *Corynebacterium* (primarily *C*. *aurimucosum* [OTU 45]), *Staphylococcus* (mainly *S*. *haemolyticus* [OTU 18] and *S*. *epidermidis* [OTU 14]) and *Enterococcus* (*E*. *faecium* [OTU 5] and *E*. *faecalis* [OTU 7]) (Fig 1 and S1 Table).

Phenotypic identification using API test system was performed on a total of 485 isolates, of which 356 (73,4%) were identified at the species level, yielding excellent (n = 31), very good (n = 123), good (n = 190) and acceptable identifications (n = 12) (Table 1). Twenty-nine OTUs (35%) could be assigned by the API test system to one species, 9 OTUs (11%) were assigned to one genus but we failed to obtain a reliable identification for 14 OTUs (16%). The remaining 32 OTUs (38%) comprised organisms with assorted identifications by the API test system at the species and/or genus level and/or as unidentified taxa (e.g. in OTU 11 [n = 2 isolates]), one isolate was identified as *Lactobacillus paracasei subsp*. *paracasei and* other strain as *Gardnerella vaginalis*) (S1 Table).

**Table 1:** Relative proportion of assignments at the taxonomic level for 16S rRNA sequencing and API test system, and proportion of agreement results between both approaches.

Identification Method	% identification at taxonomic level				Agreement between 16S rRNA and API test system at taxonomic level (%)		
	Species	Genus	Family	Unidentified	Species	Genus	Mismatched classifications
16S rRNA sequencing	92.2 (n = 447)**	7.6 (n = 37)	0.2 (n = 1)	-	-	-	-
Rapid ID 32 E	98.6 (n = 143) Ex (n = 14); VG (n = 16); G (n = 113)	0.7 (n = 1) VG (n = 1)	-	0.7 (n = 1)	64.8 (n = 94)	-	34.5 (n = 50)
Rapid ID 32A	46.0 (n = 69) Ex (n = 1); VG (n = 22); G (n = 37); Ac (n = 9)	30.4 (n = 45) Ex (n = 2); VG (n = 33); G (n = 6); Ac (n = 4)	-	23.6 (n = 34)	14.4 (n = 21)	44.5 (n = 65)	17.6 (n = 25)
Rapid ID 32 Strep	93.8 (n = 91) Ex (n = 10); VG (n = 70); G (n = 11)	6.2 (n = 6) Ex (n = 2); VG (n = 1); G (n = 3)	-	-	84.5 (n = 82)	15.5 (n = 15)	-
ID 32 GN	95.0 (n = 19) Ex (n = 3); VG (n = 4); G (n = 11); Ac (n = 1)	-	-	5.0 (n = 1)	40.0 (n = 8)	40.0 (n = 8)	15.0 (n = 3)
ID 32 Staph	61.5 (n = 16) Ex (n = 2); VG (n = 9); G (n = 4); Ac (n = 1)	38.5 (n = 10) VG (n = 1); G (n = 9)	-	-	61.5 (n = 16)	38.5 (n = 10)	-
API Coryne	32.6 (n = 16) Ex (n = 1); VG (n = 1); G (n = 13); Ac (n = 1)	55.8 (n = 22) VG (n = 22)	-	11.6 (n = 5)	16.3 (n = 7)	58.1 (n = 25)	18.6 (n = 8)
API 50 CHL	40.0 (n = 2) VG (n = 1); G (n = 1)	20.0 (n = 1) VG (n = 1)	-	40.0 (n = 2)	20.0 (n = 1)	40.0 (n = 2)	-
API 50 CHB	-	100.0 (n = 1) G (n = 1)	-	-	-	100.0 (n = 1)	-
Total API test system	73.4 (n = 356) Ex (n = 31); VG (n = 123). G (n = 190); Ac (n = 12)	17.7 (n = 86) Ex (n = 4); VG (n = 59); G (n = 19); Ac (n = 4)	-	8.9 (n = 43)	47.0 (n = 228)	25.8 (n = 125)	17.7 (n = 86)

** Values in parenthesis correspond to the number of isolates and number of isolates reported with Excellent (Ex), Very Good (VG), Good (G) and Acceptable (Ac) identifications using the miniAPI Analyser. Results reported as poor discrimination, unacceptable and not interpretable upon repeated testing were recorded as unidentified.

The closely related type strains of forty-two different OTUs determined by 16S rRNA gene analysis, were included in API system (S1 Table). Of this group, representative species of 36 OTUs included in the API database had 16S rRNA similarity values of ≥99.0% with validly named taxa, of which 18 OTUs gave congruent identifications at the species level with the identification of the API test system. Another 15 OTUs comprised isolates with different results by API test system, where some identifications at the species level were in agreement with the classification given by phylogenetic analysis. The comparison of both approaches was mainly congruent at the species and genus level. In the remaining OTUs, OTU 60 was consistent at the genus level, and OTUs 8 and 33 were found to have discrepant results between the two approaches (S1 Table).

Five of the forty-two OTUs, in which the species were included in the API system database, showed similarity values between 97.0% and 99.0% to the sequences of type strains that could not be assigned to a species identified by 16S rRNA sequence analysis. The API test system identified OTUs 2 and 63 corroborating the closely related type species classified by 16S rRNA analysis and identified OTUs 46, 57 and 58 with congruent results with the 16S rRNA analysis at the genus level while, OTU 48 was not identified by the API system.

Thirty-one OTUs with 16S rRNA similarity values above 99.0% with sequences of type strains were not included in the API database. The API test system identified four OTUs (OTU 1, 10, 32, 82) at the species level that were congruent with 16S rRNA data at genus level. Four OTUs (OTU 9, 20, 54, 62) exhibited miscellaneous results in which some identifications were consistent, at the genus level, with the phylogenetic analysis. Eleven OTUs (e.g. OTUs 23–25, 34, 35) were identified at the species or genus level by the API system in disagreement with the results of the phylogenetic analysis.

Overall, our results indicated that the Rapid ID 32 E, ID 32 GN and Rapid ID 32 Strep test systems showed the highest proportion of identifications to the species level and the lowest ratio of unidentified results (Table 1). Moreover, the highest agreement of assignments at the species level between 16S rRNA sequencing and API test system were obtained for the Rapid ID 32 Strep strip (84.5% of the isolates), followed by Rapid ID 32 E strip (64.8% of the isolates) that also showed the highest number of mismatched classifications between both methods (34.5% of the isolates) (Table 1). The high proportion of discrepant classifications for Rapid ID 32 E strip was due to OTU 80 that was phylogenetically closely related with *Enterobacter cloacae* subsp. *cloacae* (n = 48 isolates) but was generally identified as *Escherichia coli* by API test system. About 97% of the identifications at the species level by the API test system yielded quality ratings of good or above.

### Antimicrobial susceptibility profiles

Antibiotic resistance profiles were determined using ATB strips for 438 isolates belonging to 60 OTUs. For 47 strains (24 OTUs) unable to grow on ATB control wells we used the disk diffusion method.

Most of the OTUs that belonged to the class *Bacilli* (12/19 OTUs, comprising 114/133 isolates) (S2A Table) were resistant to three or more classes of antibiotics, and thus could be considered multiple antibiotic resistant (MAR) bacteria. The highest number of MAR profiles were observed among the genera *Streptococcus*, *Enterococcus*, *Lactobacillus* and *Staphylococcus*. Notably, OTU 14 comprising 8 isolates related to *Staphylococcus epidermidis* (100% homology of 16S rRNA) showed resistance patterns to 8 of the 12 different classes of antibiotics tested.

Members of the *Clostridia* were susceptible to most of the antibiotics examined (S2B Table). However, a small set of isolates were resistant to penicillin (0.5-2mg/l or 10μg), cefoxitin (16-32mg/l or 30μg) and clindamycin (2-4mg/l or 2μg). Isolates of the *Actinobacteria* (S2C Table) showed widespread sensibility to the all of the penicillins and to the antibiotics imipenem (4-8mg/l or 10μg), cefoxitin (16-32mg/l or 30μg) and chloramphenicol (8-16mg/l or 30μg). On the other hand, OTUs 38 and 39 (16 isolates) had multiple resistance phenotypes to 8 of the 14 classes of antibiotics tested. The majority of the members of the class *Bacteroidia* (S2D Table) were resistant to penicillin (0.5-2mg/l or 10μg), piperacillin (32-64mg/l or 75μg), cefotetan (16-32mg/l or 30μg) and clindamycin (2-4mg/l or 2μg). Several of the isolates related to genera *Bacteroides*, *Parabacteroides* and *Barnesiella* were resistant to 3–4 of the 10 classes of antibiotics tested. Several of the isolates belonging to the *Proteobacteria* were susceptible to antibiotics of all classes tested (S2E Table). MAR phenotypes were observed in 7 of 11 OTUs. However, only a small number of isolates that grouped in OTUs 76 (*Escherichia coli*) and 80 (*Enterobacter cloacae subsp*. *cloacae*), showed high levels of resistance to all classes of antibiotics tested, but mainly to the different penicillins examined.

## Discussion

Published reports have indicated that the overall incidence of CBU contamination varies between 0–48% [17]. Umbilical cord blood may be contaminated with microorganisms from the vaginal or perianal microbiomes and/or from skin and the environment during collection, processing and cryopreservation [17].

In this study, we analyzed 5618 CBUs under routine conditions used to identify the microorganisms isolated from BacT/ALERT prior to cryopreservation; 7.5% of the CBUs were positive for microbial contamination. A set of 485 strains were isolated and identified by both API test system and 16S rRNA gene analyses. Antimicrobial susceptibility profiles for each strain were also evaluated.

The analysis of 16S rRNA gene sequences remains the most parsimonious and cost-effective method used for the classification of organisms. Several of the surveys assessing the reliability of the identification of organisms that contaminate CBUs using 16S rRNA sequence analysis versus other phenotypic test systems, such as API have been based on the BLAST search to obtain taxonomic affiliations of the isolates. However, several reports indicate that comparisons of sequence similarities that rely on the closest BLAST hit alone, should be considered with care since it does not indicate phylogenetic relationships [18]. The 16S rRNA gene, despite being the principal support of the current taxonomic procedures used to establish microbial phylogenetic relations, is not in itself a suitable parameter for discriminative classifications because of its lack of resolution of some taxa at the species level and its analysis should be complemented with additional phenotypic properties [19,20].

In this study, we initially grouped isolates based on distinctive V1 to V5 hypervariable regions of the 16S rRNA genes taking into account the premise that each different sequence can be considered to be a different phylogenetic phylotype. Later, OTUs were created based on a similarity threshold of 99.0% of near full length 16S rRNA gene sequences. Thirty OTUs comprised organisms with different identifications by the API test system at the species and/or genus level and/or as unknown taxa, independently of the API system used, and the number of isolates present in each OTU. This inconsistency of the API identification is probably caused by intra-species phenotypic variability not predicted by the system database, and/or by unresolved phenotypic results. There are several examples of this type of discrepancy, such as OTU 5, 25, 65, 80.

Other studies that assess the contamination of CBUs indicate a lower diversity by 16S rRNA sequence analysis and/or phenotypic identification using automated test systems, probably because the number of isolates were lower or the methodologies were different [17,21–24]. These studies, as well as ours, indicate that the majority of the isolates, namely *Enterococcus spp*., *Staphylococcus spp*., *Corynebacterium spp*., *Bacteroides spp*. and members of the *Enterobacteriaceae* were primarily of perianal, skin or vaginal origin. Environmental isolates, such as *Bacillus clausii*, *Burkholderia pyrrocinia* and *Achromobacter insolitus* were rare in this study.

One major bias of the API test system is that the available databases do not include many species classified by 16S rRNA gene analysis that were isolated from the CBUs. Of the 84 OTUs defined in this study, 42 OTUs comprised species not included in the API system database. In some of these cases the API test system was not able to attribute any identification, but in 15 cases an identification at the species and/or genus level was attributed with no agreement, at the genus level, with 16S rRNA sequence analysis. However, for nine OTUs the API test system confirmed the results of 16S rRNA analysis at the genus level.

Another major problem of automated phenotypic methods is that the accuracy of the identifications is dependent on the quality of the database. In this regard, OTUs of the classes *Gammaproteobacteria* and *Bacilli*, and the family *Bacteroidaceae* were very well represented in the API database because they are the prevalent populations in clinical samples. Knowledge of the variability of the phenotypic diversity was largely increased through recent years endorsing high accuracy of identifications at the species and/or genus level. Members of the classes *Clostridia* and *Actinobacteria* were the least represented in the API database with the highest rate of unidentified or incorrect results at the species and genus level, using classifications of the 16S rRNA analysis as reference. Most members of these classes are not common clinical isolates and/or have fastidious growth conditions that are difficult to replicate under routine laboratory conditions.

Whereas the identification of all bacterial isolates is not possible or is dubious by standardized phenotypic methods, it is nearly always possible by 16S rRNA sequence analysis, because ribosomal databases cover the full extent of known taxa diversity. In most cases, phylogenetic reconstructions are not dependent on strain ecotype or individual interpretation [5]. The major limitation is the discriminating power, which is not sufficient in some genera, to permit the assignment of the strain to a group of related bacteria, which could be especially useful in clinical diagnosis. OTUs of the *Enterobacteriaceae* showed a high congruency between API test system and 16S rRNA analysis, with the exception of OTU 80 that was phylogenetically most closely related to *Enterobacter cloacae* subsp. *cloacae* (99.7% 16S rRNA identity) but was mainly identified as *Escherichia coli* by API test system with the manufacturer’s result of “good species identification”. OTU 80 shares an average 16S rRNA gene sequence similarity of 96% with *Escherichia coli* ATCC 11775^T^, indicating that OTU 80 is not phylogenetically closely related to isolates of *Escherichia coli* [13]. Other studies showed that identifications below “very good” with the API/VITEK test systems should be considered less reliable and further analysis should be performed [25,26].

In other cases, the application of supplemental phenotypic tests could be helpful to support the molecular identification. Some examples are OTUs 2 and 63, in which the representative isolate has a sequence identify below 99.0% to the most closely related reference strain. The agreement between API test system and the phylogenetic assignment permits the identification of these strains at the species level.

The need for antibiotic susceptibility testing of clinical isolates has been debated, although there is little doubt that susceptibility results are important for the selection of therapeutic agents in case of CBU application. While we did not use the same antibiotic susceptibility set for each group in an attempt to follow the manufacturer’s recommendations, the results show that some groups had higher antibiotic resistance patterns than others. In general, isolates affiliated to the classes *Bacilli* and *Bacteroidia* showed the highest multi-resistance indices and microorganisms of the *Clostridia* displayed the most antibiotic sensitive phenotypes. This analysis showed that the majority of the OTUs (60%), have similar resistance patterns within the same OTU while thirty-four OTUs did not exhibit identical antibiotic resistance phenotypes within each OTU. These results are in agreement with other studies that show that clinical isolates possess a large variety of antibiotic resistances [27–30]. The majority of the organisms isolated in this study are related to clinical isolates and reflect populations that originate from vaginal, anal or perianal areas, some of which could cause disease under the appropriate conditions. Moreover, the guidelines for stem cell preservation from umbilical cord blood recommend that antibiotic susceptibilities of contaminant bacteria be evaluated to ensure suitable utilization of antibiotics treatment with these stem cells [3].

Altogether the results showed that members of the *Enterococcus spp*., *Staphylococcus spp*., *Corynebacterium spp*., *Bacteroides spp*. and of the *Enterobacteriaceae* were the prevalent taxa most prone to contaminate cord blood units. 16S rRNA sequencing analysis could unambiguously perform the assignment of the isolates to closely related species. Fifty percent of the OTUs determined by 16S analysis were included in the API databases. The API test system Rapid ID 32 Strep and Rapid ID 32 E showed the highest proportion of identifications to the species level, the lowest ratio of unidentified results, and the highest agreement to the results of 16S rRNA assignments. The antibiotic resistance profiles of the isolates showed it to be a valuable source of information for the proper application of cryopreserved stem cell that could suffer microbial contamination during the collection and processing procedures of CBUs.

## Supporting Information

S1 Fig Rarefaction analysis for the observed number of OTUs in the isolates dataset. (PDF)

S2 Fig Maximum likelihood tree showing the phylogenetic relationships of the 16S rRNA gene sequences of the isolates with the most closely related reference sequences of the LTP database 115. (PDF)

S1 Table Relative abundance and identification by 16S rRNA sequencing and API test system of the OTUs. (PDF)

S2 Table Antibiotic resistance patterns observed for OTUs belonging to the *Bacilli* (S2A), *Clostridia* (S2B), *Actinobacteria* (S2C), *Bacteroidia* (S2D) and *Proteobacteria* (S2E). (XLSX)
