# Supplementary figures and images for: Microbial Contaminants of Cord Blood Units Identified by 16S rRNA Sequencing and by API Test System, and Antibiotic Sensitivity Profiling
Source: PLoS One. 2015 Oct 29;10(10):e0141152. doi: 10.1371/journal.pone.0141152 (PMC4626235; doi:10.1371/journal.pone.0141152)

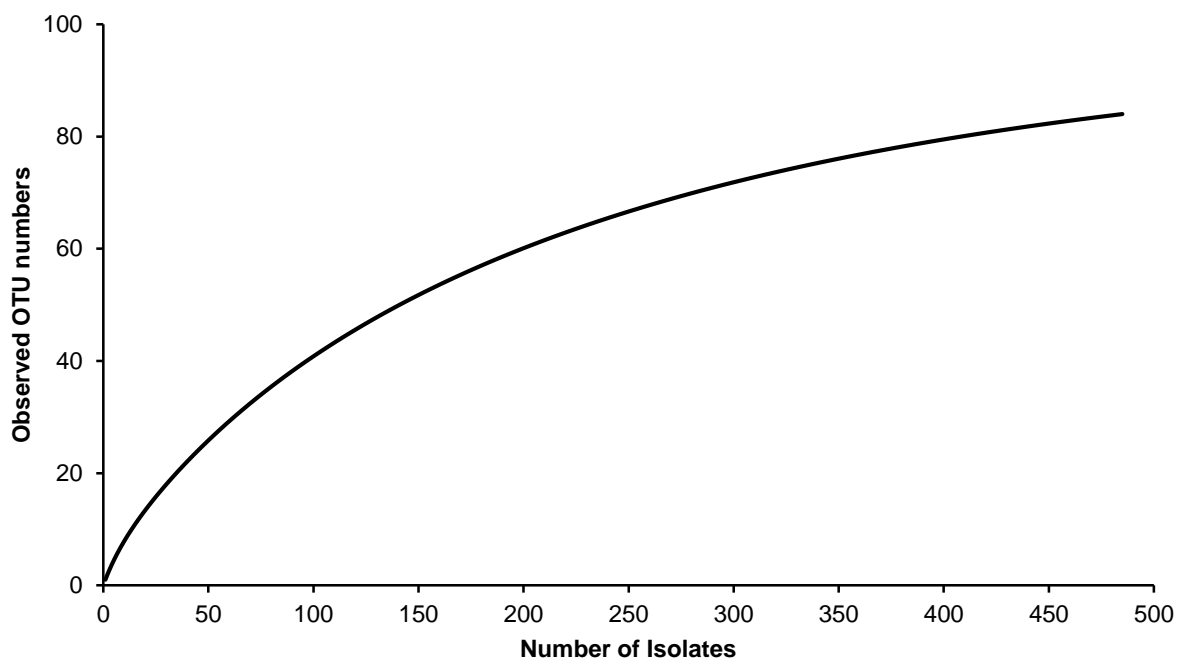

**S1 Fig.** Rarefaction analysis for the observed number of OTUs in the isolates dataset.

Supplement: S1 Fig — (PDF) [file pone.0141152.s001.pdf]
