# Supplementary material for: Microbial Contaminants of Cord Blood Units Identified by 16S rRNA Sequencing and by API Test System, and Antibiotic Sensitivity Profiling
Source: PLoS One. 2015 Oct 29;10(10):e0141152. doi: 10.1371/journal.pone.0141152 (PMC4626235; doi:10.1371/journal.pone.0141152)
Supplement: S2 Fig — (PDF) [file pone.0141152.s002.pdf]

S2 Fig. (Continued)

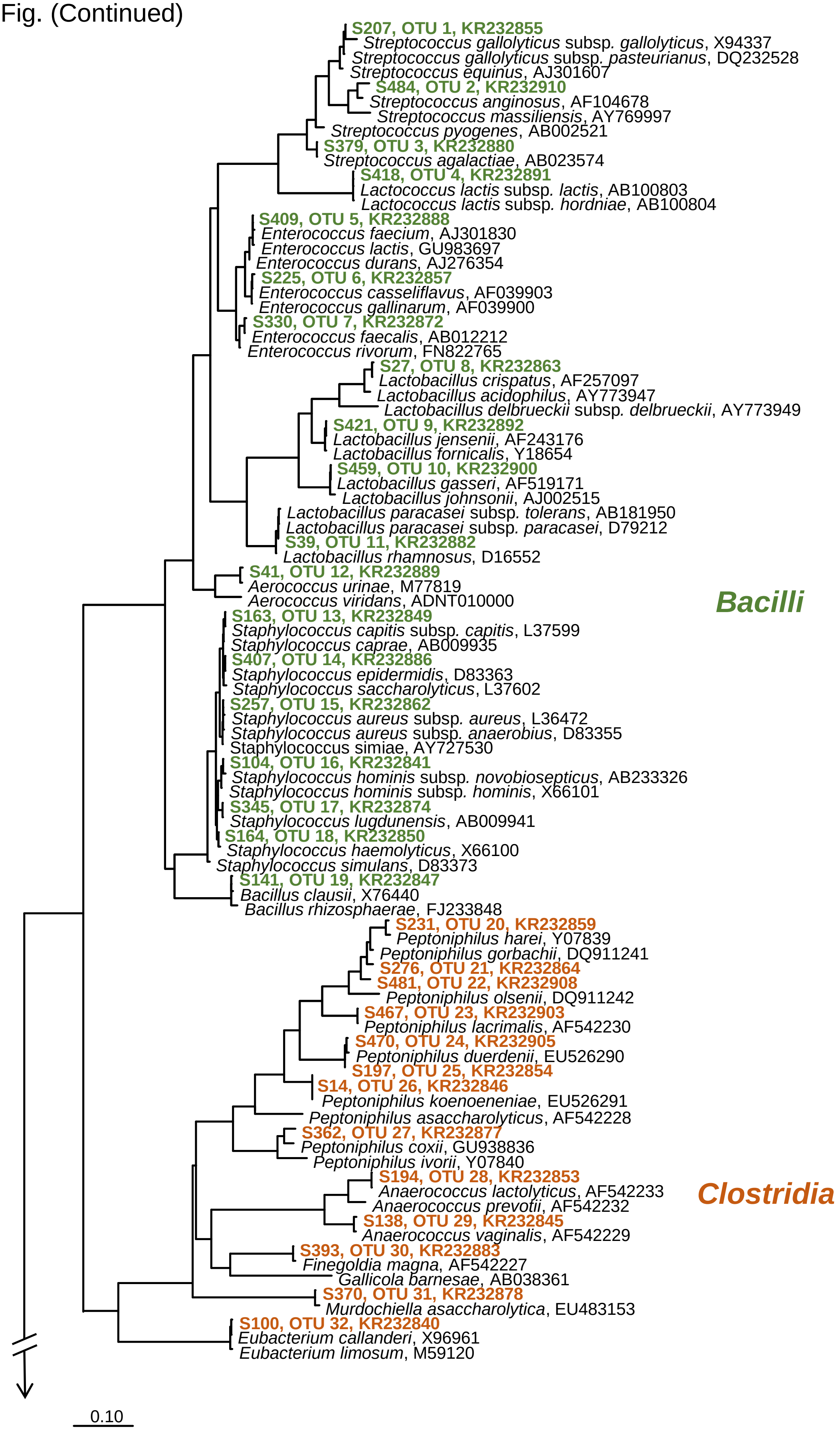

S2 Fig. (Continued)

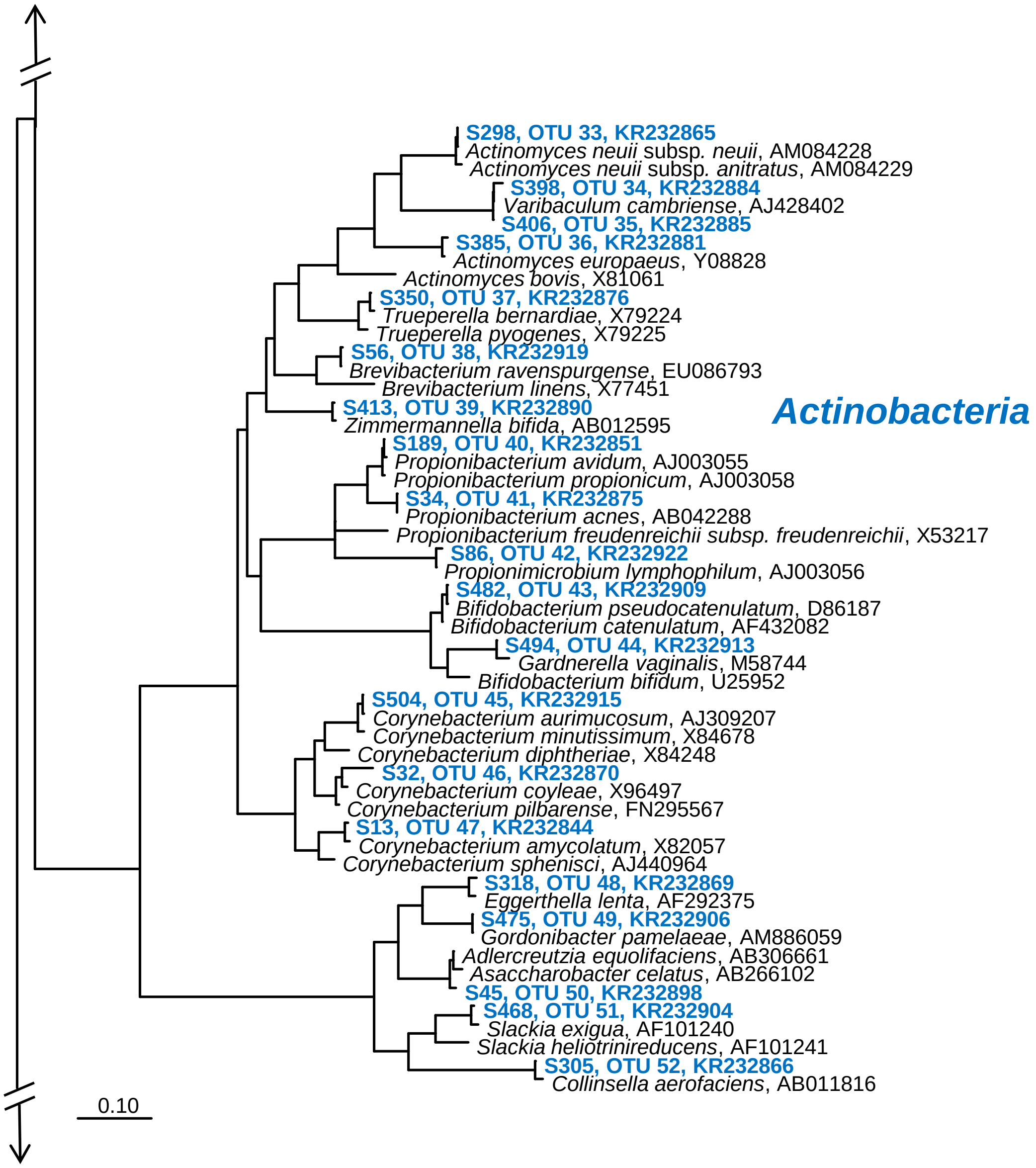

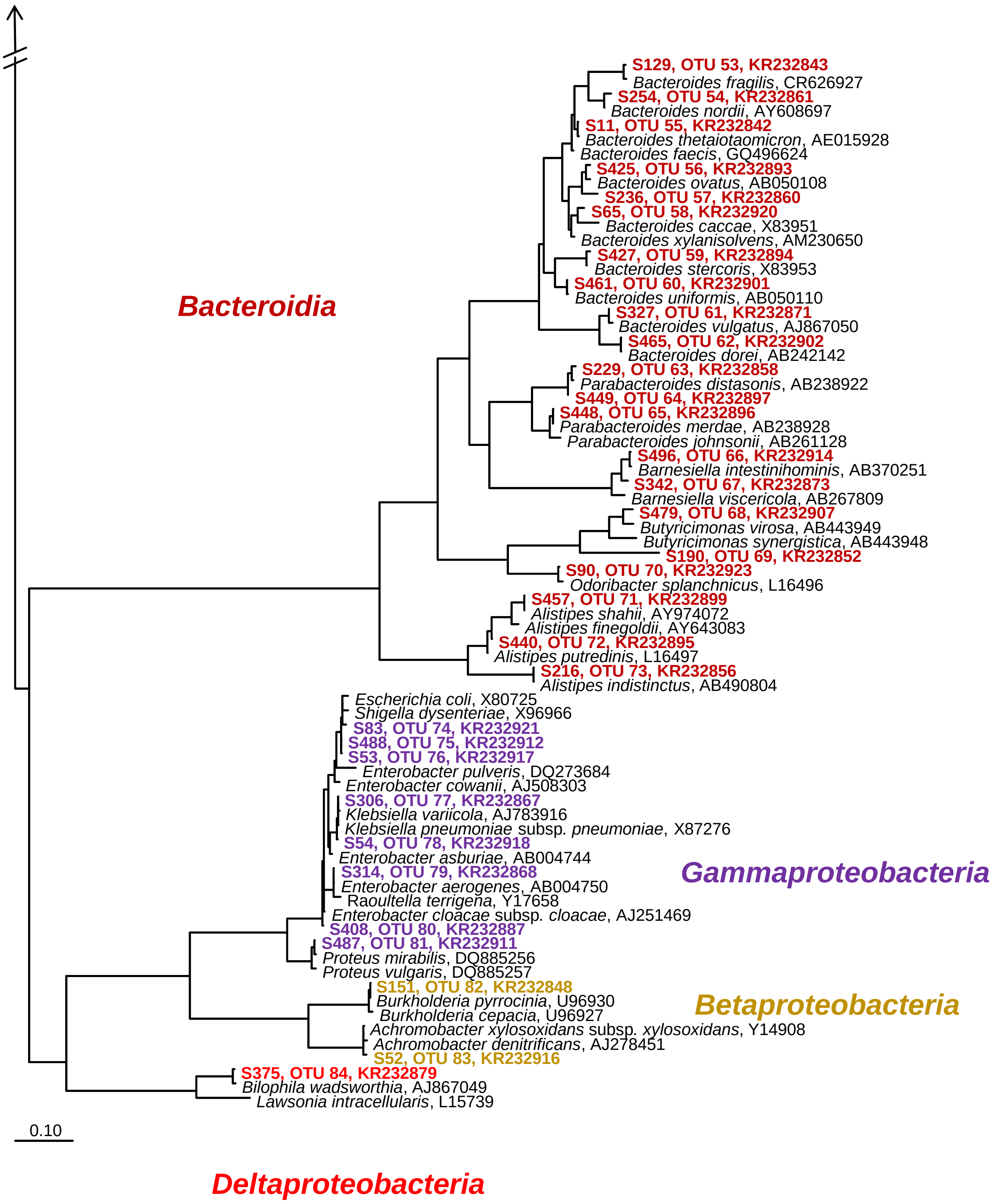

S2 Fig. Maximum likelihood tree showing the phylogenetic relationships of the 16S rRNA gene sequences of the isolates with the most closely related reference sequences of the LTP database 115. Tree was constructed using sequences longer than 1270 bp with a 20% conservational filter. The scale bar infers 10 nucleotide substitutions per 100 sequenced nucleotides.
