# Supplementary material for: Microbial Contaminants of Cord Blood Units Identified by 16S rRNA Sequencing and by API Test System, and Antibiotic Sensitivity Profiling
Source: PLoS One. 2015 Oct 29;10(10):e0141152. doi: 10.1371/journal.pone.0141152 (PMC4626235; doi:10.1371/journal.pone.0141152)
Supplement: S1 Table — (PDF) [file pone.0141152.s003.pdf]

S1 Table. Relative abundance and identification by 16S rRNA sequencing and API test system of the OTUs.

OTU	Representative strain, accession number	Closest relative, accession number	Closest relative identity (%)	*	Identification by API test system		Relative abundance (%)	Taxonomic afiliation (Class level)
1	S207, KR232855	*Streptococcus gallolyticus subsp. pasteurianus, DQ232528*	99.8	□	*Streptococcus equinus* (Excellent n=2; Good n=1)	🟡	0.6 (n=3)	*Bacillii*
2	S484, KR232910	*Streptococcus anginosus, AF104678*	**98.3**	■	*Streptococcus anginosus* (Excellent n=2)	🟡	0.4 (n=2)	
3	S379, KR232880	*Streptococcus agalactiae, AB023574*	100.0	■	*Streptococcus agalactiae* (Excellent n=1; Very good n=7; Good n=1); *Streptococcus sp.* (Good at the genus level n=1)	🟡	2.1 (n=10)	
4	S418, KR232891	*Lactococcus lactis subsp. lactis, AB100803*	100.0	■	*Lactococcus lactis subsp. lactis* (Excellent n=3)	🟢	0.6 (n=3)	
5	S409, KR232888	*Enterococcus faecium, AJ301830*	99.9	■	*Enterococcus faecium* (Good n=7); *Enterococcus durans* (Very good n=3); *Enterococcus sp.* (Excellent at the Genus level n=2); *Enterococcus faecalis* (Good n=1)	🟡	2.7 (n=13)	
6	S225, KR232857	*Enterococcus casseliflavus, AF039903*	99.8	■	*Enterococcus casseliflavus* (Good n=1)	🟢	0.2 (n=1)	
7	S330, KR232872	*Enterococcus faecalis, AB012212*	99.8	■	*Enterococcus faecalis* (Excellent n=1; Very good n=60; Good n=1); Enterococcus sp. (Very good at the genus level n=1; Good at the genus level n=1)	🟡	13.2 (n=64)	
8	S27, KR232863	*Lactobacillus crispatus, AF257097*	99.9	■	Unidentified	🔴	0.4 (n=2)	
9	S421, KR232892	*Lactobacillus jensenii, AF243176*	100.0	□	*Gemella morbillorum* (Very good n=2); *Lactobacillus sp.* (Very good at the genus level n=1)	⚪	0.6 (n=3)	
10	S459, KR232900	*Lactobacillus gasseri, AF519171*	100.0	□	*Lactobacillus acidophilus* (Very good n=1; Good n=1)	🟡	0.4 (n=2)	
11	S39, KR232882	*Lactobacillus paracasei subsp. paracasei, D79212*	100.0	■	*Lactobacillus paracasei subsp. Paracasei* (Good n=1); *Gardnerella vaginalis* (Very good n=1)	⚪	0.4 (n=2)	
12	S41, KR232889	*Aerococcus urinae, M77819*	99.2	■	*Aerococcus urinae* (Excellent n=1)	🟢	0.2 (n=1)	
13	S163, KR232849	*Staphylococcus capitis subsp. capitis, L37599*	100.0	■	*Staphylococcus capitis* (Excellent n=1; Very good n=1)	🟢	0.4 (n=2)	
14	S407, KR232886	*Staphylococcus epidermidis*, D83363	100.0	■	*Staphylococcus sp.* (Very good at the genus level n=1; Good at the genus level n=6); *Staphylococcus epidermidis* (Good n=1; Acceptable n=1)	🟡	1.9 (n=9)	
15	S257, KR232862	*Staphylococcus aureus subsp. aureus, L36472*	100.0	■	*Staphylococcus aureus* (Excellent n=1; Very good n=1; Good n=1)	🟢	0.6 (n=3)	
16	S104, KR232841	*Staphylococcus hominis subsp. hominis*, X66101	99.9	■	*Staphylococcus sp.* (Good at the genus level n=3); *Staphylococcus simulans* (Good n=1)	🟡	0.8 (n=4)	
17	S345, KR232874	*Staphylococcus lugdunensis*, AB009941	100.0	■	*Staphylococcus lugdunensis* (Very good n=2)	🟢	0.4 (n=2)	
18	S164, KR232850	*Staphylococcus haemolyticus,* X66100	99.8	■	*Staphylococcus haemolyticus* (Very good n=5; Good n=1)	🟢	1.2 (n=6)	
19	S141, KR232847	*Bacillus clausii,* X76440	100.0	□	*Bacillus sp.* (Good at the genus level n=1)	🟡	0.2 (n=1)	
20	S231, KR232859	*Peptoniphilus harei,* Y07839	99.0	□	*Peptoniphilus asaccharolyticus* (Very good n=1; Good n=6); Unidentified (n=1)	⚪	1.6 (n=8)	*Clostridia*
21	S276, KR232864	*Peptoniphilus gorbachii,* DQ911241	**98.4**	□	*Peptoniphilus asaccharolyticus* (Good n=3); Unidentified (n=1)	⚪	0.8 (n=4)	
22	S481, KR232908	*Peptoniphilus gorbachii,* DQ911241	**97.5**	□	*Peptoniphilus asaccharolyticus* (Good n=1)	🟡	0.2 (n=1)	
23	S467, KR232903	*Peptoniphilus lacrimalis,* AF542230	100.0	□	*Gemella morbillorum* (Good n=1)	🔴	0.2 (n=1)	
24	S470, KR232905	*Peptoniphilus duerdenii*, EU526290	99.1	□	*Finegoldia magna* (Very good n=1); Unidentified (n=1)	🔴	0.4 (n=2)	
25	S197, KR232854	*Peptoniphilus duerdenii*, EU526290	99.1	□	*Anaerococcus prevotii* (Good n=1); *Finegoldia magna* (Good n=1); Unidentified (n=1)	🔴	0.6 (n=3)	
26	S14, KR232846	*Peptoniphilus koenoeneniae,* EU526291	99.9	□	Unidentified	🔴	0.2 (n=1)	
27	S362, KR232877	*Peptoniphilus coxii,* GU938836	**96.3**	□	*Clostridium sp.* (Good at the genus level n=1)	🔴	0.2 (n=1)	
28	S194, KR232853	*Anaerococcus lactolyticus,* AF542233	99.9	□	Unidentified	🔴	0.4 (n=2)	
29	S138, KR232845	*Anaerococcus vaginalis,* AF542229	**98.8**	□	Unidentified	🔴	0.2 (n=1)	
30	S393, KR232883	*Finegoldia magna,* AF542227	99.7	■	*Anaerococcus prevotii* (Good n=2); *Finegoldia magna* (Very good n=1); Unidentified (n=1)	⚪	0.8 (n=4)	
31	S370, KR232878	*Murdochiella asaccharolytica,* EU483153	99.3	□	Unidentified	🔴	0.4 (n=2)	
32	S100, KR232840	*Eubacterium callanderi,* X96961	99.9	□	*Eubacterium limosum* (Good n=1)	🟡	0.2 (n=1)	
33	S298, KR232865	*Actinomyces neuii subsp. neuii,* AM084228	100.0	■	*Cellulomonas sp./Microbacterium sp.* (Excellent n=1; Good n=1)	🔴	0.4 (n=2)	*Actinobacteria*
34	S398, KR232884	*Varibaculum cambriense,* AJ428402	99.0	□	*Gardnerella vaginalis* (Good n=2); Unidentified (n=1)	🔴	0.6 (n=3)	
35	S406, KR232885	*Varibaculum cambriense,* AJ428402	99.9	□	*Peptostreptococcus anaerobius* (Very good n=1; Good n=1)	🔴	0.4 (n=2)	
36	S385, KR232881	*Actinomyces europaeus,* Y08828	**98.8**	□	*Gardnerella vaginalis* (Good n=3); *Propionibacterium propionicus* (Good n=1)	🔴	0.8 (n=4)	
37	S350, KR232876	*Trueperella bernardiae,* X79224	99.6	□	*Gardnerella vaginalis* (Good n=2)	🔴	0.4 (n=2)	
38	S56, KR232919	*Brevibacterium ravenspurgense,* EU086793	99.8	▨	*Corynebacterium propinguum* (Good n=1); *Corynebacterium afermentans/coyleae* (Acceptable n=1); Unidentified (n=1)	🔴	0.6 (n=3)	
39	S413, KR232890	*Zimmermannella bifida,* AB012595	99.6	□	Unidentified	🔴	0.2 (n=1)	
40	S189, KR232851	*Propionibacterium avidum,* AJ003055	99.8	■	*Propionibacterium avidum* (Excellent n=1; Very good n=1)	🟢	0.4 (n=2)	
41	S34, KR232875	*Propionibacterium acnes,* AB042288	100.0	■	*Propionibacterium acnes* (Very good n=1; Good n=2); Unidentified (n=1)	⚪	0.8 (n=4)	
42	S86, KR232922	*Propionimicrobium lymphophilum,* AJ003056	99.5	□	Unidentified	🔴	0.2 (n=1)	
43	S482, KR232909	*Bifidobacterium pseudocatenulatum,* D86187	99.8	▨	*Bifidobacterium sp.* (Very good n=1)	🟡	0.2 (n=1)	
44	S494, KR232913	*Gardnerella vaginalis,* M58744	99.0	■	*Gemella morbillorum* (Good n=1); *Actinomyces meyeri* (Good n=1); *Gardnerella vaginalis* (Good n=1)	⚪	0.6 (n=3)	
45	S504, KR232915	*Corynebacterium aurimucosum,* AJ309207	100.0	□	*Corynebacterium sp.* (Very good at the genus level n= 13); *Corynebacterium striatum/amycolatum* (Good n=1); Unidentified (n=1)	🟡	3.1 (n=15)	
46	S32, KR232870	*Corynebacterium coyleae, X96497*	**97.1**	□	*Corynebacterium sp.* (Very good at the genus level n=5)	🟡	1.0 (n=5)	
47	S13, KR232844	*Corynebacterium amycolatum*, X82057	99.2	■	*Corynebacterium sp.* (Very good at the genus level n=4); *Corynebacterium jeikeium* (Good n=1); *Corynebacterium striatum/amycolatum* (Good n=1)	🟡	1.2 (n=6)	
48	S318, KR232869	*Eggerthella lenta,* AF292375	**98.5**	■	Unidentified	🔴	1.2 (n=6)	
49	S475, KR232906	*Gordonibacter pamelaeae,* AM886059	100.0	□	*Clostridium sp.* (Acceptable at the genus level n=1); Unidentified (n=1)	🔴	0.4 (n=2)	
50	S45, KR232898	*Adlercreutzia equolifaciens,* AB306661	99.3	□	Unidentified	🔴	0.2 (n=1)	
51	S468, KR232904	*Slackia exigua,* AF101240	99.0	□	*Gemella morbillorum* (Good n=1); Unidentified (n=1)	🔴	0.4 (n=2)	
52	S305, KR232866	*Collinsella aerofaciens,* AB011816	99.1	□	*Peptostreptococcus anaerobius* (Acceptable n=2); *Eggerthella lenta* (Good n=1)	🔴	0.6 (n=3)	
53	S129, KR232843	*Bacteroides fragilis,* CR626927	99.6	■	*Bacteroides fragilis* (Very good n=3; Good n=1)	🟢	0.8 (n=4)	*Bacteroidia*
54	S254, KR232861	*Bacteroides nordii,* AY608697	99.3	□	*Bacteroides sp.* (Very good at the Genus level n=1)	🟡	0.2 (n=1)	
55	S11, KR232842	*Bacteroides thetaiotaomicron*, AE015928	**95.4**	■	*Bacteroides thetaiotaomicron* (Very good n=2)	🟢	0.4 (n=2)	
56	S425, KR232893	*Bacteroides ovatus,* AB050108	99.0	■	*Bacteroides ovatus* (Good n=1)	🟢	0.2 (n=1)	
57	S236, KR232860	*Bacteroides ovatus,* AB050108	**97.3**	■	*Bacteroides sp.* (Good at the genus level n=1)	🟡	0.2 (n=1)	
58	S65, KR232920	*Bacteroides caccae,* X83951	**97.0**	■	*Bacteroides sp.* (Excellent at the genus level n=2; Very good at the Genus level n=1; Good at the genus level n=1); *Bacteroides eggerthii* (Very good n=1)	🟡	1.0 (n=5)	
59	S427, KR232894	*Bacteroides stercoris,* X83953	99.3	■	*Bacteroides stercoris* (Good n=1)	🟢	0.2 (n=1)	
60	S461, KR232901	*Bacteroides uniformis,* AB050110	99.8	■	*Bacteroides sp.* (Very good at genus level n=26; Good at the genus level n=1; Acceptable at the Genus level n=1)	🟡	5.8 (n=28)	
61	S327, KR232871	*Bacteroides vulgatus,* AJ867050	99.4	■	*Bacteroides vulgatus* (Very good n=1; Acceptable n=6)	🟢	1.4 (n=7)	
62	S465, KR232902	*Bacteroides dorei,* AB242142	100.0	□	*Parabacteroides merdae* (Acceptable n=1); Unidentified (n=1); *Bacteroides vulgatus* (Very good n=1)	⚪	0.6 (n=3)	
63	S229, KR232858	*Parabacteroides distasonis,* AB238922	**98.7**	■	*Parabacteroides distasonis* (Excellent n=1; Very good n=1; Good n=1)	🟢	0.6 (n=3)	
64	S449, KR232897	Parabacteroides distasonis, AB238922	99.3	■	*Parabacteroides distasonis* (Very good n=1; Good n=2)	🟢	0.6 (n=3)	
65	S448, KR232896	*Parabacteroides merdae,* AB238928	100.0	■	*Parabacteroides distasonis* (Good n=1); *Parabacteroides merdae* (Good n=1); *Bacteroides sp.* (Very good at the genus level n=1)	⚪	0.6 (n=3)	
66	S496, KR232914	*Barnesiella intestinihominis,* AB370251	99.5	□	*Bacteroides capillosus* (Very good n=3; Good n=2); *Bacteroides sp.* (Good at the genus level n=1; Acceptable at the genus level n=1)	🔴	1.4 (n=7)	
67	S342, KR232873	*Barnesiella intestinihominis,* AB370251	**98.2**	□	*Prevotella sp.* (Good at the genus level n=1)	🔴	0.2 (n=1)	
68	S479, KR232907	*Butyricimonas virosa,* AB443949	**97.0**	□	Unidentified	🔴	0.2 (n=1)	
69	S190, KR232852	*Butyricimonas virosa,* AB443949	**90.3**	□	Unidentified	🔴	0.2 (n=1)	
70	S90, KR232923	*Odoribacter splanchnicus,* L16496	99.3	□	Unidentified	🔴	0.8 (n=4)	
71	S457, KR232899	*Alistipes shahii,* AY974072	99.5	□	*Bacteroides sp*. (Very good at the genus level n=4; Acceptable at the genus level n=1)	🔴	1.0 (n=5)	
72	S440, KR232895	*Alistipes putredinis,* L16497	99.5	□	*Prevotella intermedia* (Good n=1); Unidentified (n=3)	🔴	0.8 (n=4)	
73	S216, KR232856	*Alistipes indistinctus,* AB490804	100.0	□	Unidentified	🔴	0.2 (n=1)	
74	S83, KR232921	*Escherichia coli*, X80725	99.3	■	*Escherichia coli* (Excellent n=1)	🟢	0.2 (n=1)	*γ-Proteobacteria*
75	S488, KR232912	*Escherichia coli,* X80725	99.8	■	*Escherichia coli* (Excellent n=1; Very good n=2; Good n=1)	🟢	0.8 (n=4)	
76	S53, KR232917	*Escherichia coli,* X80725	99.8	■	*Escherichia coli* (Excellent n=1; Very good n=1; Good n=77); *Enterobacter cloacae* (Excellent n=1)	⚪	16.5 (n=80)	
77	S306, KR232867	*Klebsiella pneumoniae subsp. pneumoniae,* X87276	99.6	■	*Klebsiella pneumoniae subsp. pneumoniae* (Excellent n=1; Very good n=1; Good n=6); Unidentified (n=1)	⚪	1.9 (n=9)	
78	S54, KR232918	*Klebsiella pneumoniae subsp. pneumoniae*, *X87276*	99.5	■	*Klebsiella pneumoniae subsp. pneumoniae* (Good n=2)	🟢	0.4 (n=2)	
79	S314, KR232868	*Enterobacter aerogenes,* AB004750	100.0	■	*Enterobacter aerogenes* (Excellent n=1); *Raoultella terrigena* (Good n=1)	⚪	0.4 (n=2)	
80	S408, KR232887	*Enterobacter cloacae subsp. cloacae*, AJ251469	99.7	■	*Escherichia coli* (Excellent n=5; Very good n=15; Good n=30); *Enterobacter sp.* (Very good at the genus level n=1); Unidentified (n=1)	⚪	10.7 (n=52)	
81	S487, KR232911	*Proteus mirabilis,* DQ885256	99.8	■	*Proteus mirabilis* (Excellent n=4)	🟢	0.8 (n=4)	
82	S151, KR232848	*Burkholderia pyrrocinia*, U96930	100.0	□	*Burkholderia cepacia* (Excellent n=1; Very good n=1; Good n=5; Acceptable n=1)	🟡	1.6 (n=8)	*β-Proteobacteria*
83	S52, KR232916	*Achromobacter xylosoxidans subsp. xylosoxidans*, Y14908	99.4	■	*Achromobacter xylosoxidans* (Good n=3)	🟢	0.6 (n=3)	
84	S375, KR232879	*Bilophila wadsworthia*, AJ867049	99.4	□	Unidentified	🔴	0.4 (n=2)	*δ-Proteobacteria*

Phylogenetic affiliation of the 16S rRNA genes and the similarity value to the closest reference sequence in LTP 115 database are shown. Identity values below 99.0% are marked in red. Identification and quality result by API test system are shown. Different identifications for the same OTU can occur by API identification. Agreement results between 16S rRNA sequencing and API test system are marked with green circles for congruent results at the species level, yellow circles for congruent or mixed results at the genus level, grey circles for OTUs with congruent results at the species or genus level mixed with non-congruent or unclassified results, red circles for non-congruent at the genus level or unclassified results.

*, Black boxes indicate if OTUs classified by 16S rRNA sequencing belong to species included in the API test system, grey boxes if API test system is able to detect the species at the genus level and white boxes if OTUs belong to species not included in API database.
